# Scion control of miRNA abundance and tree maturity in grafted avocado

**DOI:** 10.1186/s12870-019-1994-5

**Published:** 2019-09-03

**Authors:** Muhammad Umair Ahsan, Alice Hayward, Mobashwer Alam, Jayeni Hiti Bandaralage, Bruce Topp, Christine Anne Beveridge, Neena Mitter

**Affiliations:** 1 Queensland Alliance for Agriculture and Food Innovation, The University of Queensland, St. Lucia, Brisbane, Queensland 4072 Australia; 2 School of Biological Sciences, The University of Queensland, St. Lucia, Brisbane, Queensland 4072 Australia

**Keywords:** miR156, miR172, *SPL*, Juvenility, Grafting, Scion, Rootstock, Graft-transmissibility, Avocado, *TPS1*

## Abstract

**Background:**

Grafting is the common propagation method for avocado and primarily benefits orchard production by reducing the time to tree productivity. It also allows use of scions and rootstocks specifically selected for improved productivity and commercial acceptance. Rootstocks in avocado may be propagated from mature tree cuttings (‘mature’), or from seed (‘juvenile’). While the use of mature scion material hastens early bearing/maturity and economic return, the molecular factors involved in the role of the scion and/or rootstock in early bearing/reduced juvenility of the grafted tree are still unknown.

**Results:**

Here, we utilized juvenility and flowering associated miRNAs; miR156 and miR172 and their putative target genes to screen pre-graft and post-graft material in different combinations from avocado. The abundance of mature miR156, miR172 and the miR156 target gene *SPL4*, showed a strong correlation to the maturity of the scion and rootstock material in avocado. Graft transmissibility of miR156 and miR172 has been explored in annual plants. Here, we show that the scion may be responsible for grafted tree maturity involving these factors, while the rootstock maturity does not significantly influence miRNA abundance in the scion. We also demonstrate that the presence of leaves on cutting rootstocks supports graft success and contributes towards intergraft signalling involving the carbohydrate-marker *TPS1*.

**Conclusion:**

Here, we suggest that the scion largely controls the molecular ‘maturity’ of grafted avocado trees, however, leaves on the rootstock not only promote graft success, but can influence miRNA and mRNA abundance in the scion. This constitutes the first study on scion and rootstock contribution towards grafted tree maturity using the miR156-*SPL4*-miR172 regulatory module as a marker for juvenility and reproductive competence.

**Electronic supplementary material:**

The online version of this article (10.1186/s12870-019-1994-5) contains supplementary material, which is available to authorized users.

## Background

Grafting is an ancient technique widely used for crop improvement in agriculture. It is a process in which two compatible plants are combined using a bud or branch from one plant (scion) and the roots of another plant (rootstock). In fruit trees like avocado, grafting provides a dual plant system to increase orchard productivity. Usually, scions with high yield and quality are grafted onto rootstocks with improved stress/disease tolerance. In this dual plant system, rootstocks are also selected to increase orchard efficiency through vigour control and yield improvement of the scion. Increasing precocity is another benefit of grafting, as scions taken from mature trees show significantly earlier bearing and maturity relative to trees grown from seed. Many tree crops with long juvenile phases are grown as grafted plants to obtain faster return on investment.

Grafting success requires sufficient healing of the union to allow biomolecular signalling and nutrient transportation between root and shoot, an essential system to maintain growth and survival of both organs. This includes transportation of photosynthates (carbon), ions, water, nutrients, hormones and proteins/amino acids within the phloem and xylem vasculatures. Signalling in the xylem is unidirectional from root to shoot, however, bi-directional signalling can be carried out by phloem, which comprises specialized vascular bundles connected through plasmodesmata (pores). More recently, these pores have been shown to facilitate long-distance signalling of regulatory molecules including protein/RNA [1].

*Arabidopsis* and *Nicotiana benthamiana* heterografts have revealed that over one hundred mRNA transcripts may move across the graft union [2]. This movement of mRNAs can occur both from shoot to root as well as from root to shoot [3]. This phenomenon was also observed in grapevine, which is a perennial plant [4]. In addition to mRNA transcripts, grafting experiments also revealed mobility of small RNA (smRNAs) molecules via phloem to reach specific tissues [5]. Of these smRNAs, microRNAs (miRNAs) were documented to be involved in scion/rootstock signalling in regulating biotic and abiotic factors such as drought stress and tuberization [6–9].

miRNAs are 20–24 nucleotide (nt) long, non-coding RNA molecules that negatively regulate gene activity by targeting specific gene transcripts for degradation. Interestingly, key plant miRNAs that are known to be involved in the juvenile-adult phase transition, miR156 and miR172, are shown to be graft transmissible in potato [9, 10]. Sequential activity of miR156 and miR172 mediates the juvenile-to-adult transition in perennials as well as in annuals by downregulating members of the *SQUAMOSA promoter binding protein-like (SPL)* and *APETALA2-like* transcription factor gene families, respectively [11–16]. Overexpression of miR156 results in delays in flowering while miR172 overexpression results in the opposite phenotype. In juvenile plants miR156 is highly abundant, which indirectly suppresses miR172, while mature trees have low miR156 levels and high miR172 levels. We have previously shown that this model holds true for both miR156 and miR172 abundance in avocado [17].

It has been suggested that a mobile signal(s) orginating from leaves regulates plant phase change, involving repression of miR156 [18]. Defoliation experiments in *Arabidopsis* and *Nicotiana benthamiana* resulted in a prolonged juvenile phase with an increased level of miR156. Sugar signals can be thought of as a potential candidate for this mobile regulation [19, 20]. Recently, it has been suggested that trehalose-6-phosphate (T6P) regulates flowering in the shoot apical meristem and in the leaves [21]. Further, *Trehalose-6-Phosphate Synthase 1* (*TPS1*), a key enzyme in the T6P pathway which regulates carbohydrate availability, functions upstream of florigen *FT* in the age-dependent flowering pathway in leaves [22].

Avocado rootstocks for grafting can be derived via seeds or via clonal propagation by rooting cuttings taken from mature trees. In the case of clonal rootstocks, this material is presumed physiologically mature as it derived from a mature tree cutting and subjected to rooting induction. Meanwhile, seedlings are physiologically juvenile.

As grafting on seedling or clonal rootstocks is widely used in avocado for commercial propagation, we questioned if the presumed difference in physiological maturity of these rootstocks is reflected in the abundance of these miRNAs. Given the proposed graft-transmissibility of miR156 and miR172 in other species, we also questioned if the rootstock origin could influence miRNA levels and their target genes in the scion post-grafting. In avocado, grafted trees usually start flowering the next season after planting if conditions are favourable, regardless of rootstock. This observation suggests that the scion maturity determines the maturity level of the grafted plant. However, it has also been suggested that clonal avocado rootstocks may promote earlier flowering and fruiting than seedling rootstocks as they are derived from mature tissues, but this has not been formally examined [23]. In this study, we hypothesized that the adult trait signals in the mature scion overcomes any potential juvenility factors associated with seedling rootstocks and the resultant grafted plants behave like a plant in reproductive phase. We show significant differences in abundances of miR156 and miR172 between seedling and cutting rootstocks, but this had limited impact on the miRNAs and target gene abundance in the mature budwood scion. Instead, we reveal that the scion is mainly responsible for mature miR156 and miR172 abundance in this woody tree crop, and the maturity of the scion thus reflects the abundance of these miRNAs locally. We do, however, show evidence for some long-distance signalling that may involve leaves below the graft union, regulating the carbohydrate maker gene *TPS1* and these miRNAs.

## Results

In order to quantify the abundance of phase-change associated miRNAs (miR156 and miR172) in seedling versus clonal rootstocks and detect any possible root-to-shoot signalling in avocado, grafting experiments were designed. Scions comprising either the primary growing shoot of young seedlings or budwood from mature age trees (mature scionwood) were grafted onto both seedling and cutting (clonal) rootstocks to determine the relative influence of scion vs rootstock maturity on mature microRNA and gene expression in grafted avocado scions.

### Assessment of pre-graft maturity

**Fig. 1:**
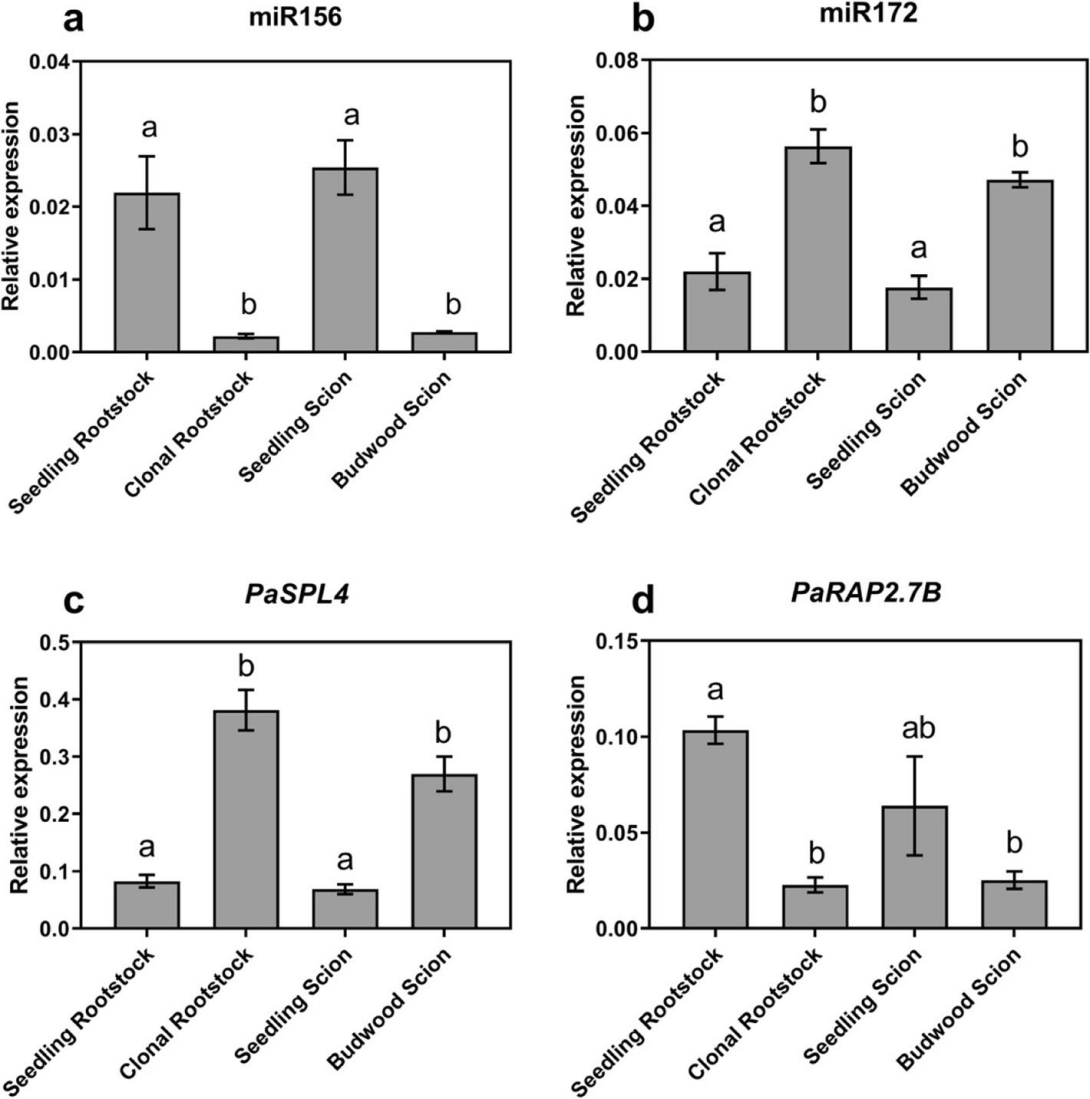
Transcript abundance of miRNAs and their putative target genes in pre-graft avocado material. Expression analysis (qRT-PCR) of **a** miR156, **b** miR172, **c** *PaSPL4* and **d** *PaRAP2.7B* in pre-graft avocado leaves sampled from the seedling rootstock, clonal rootstock, seedling scion and budwood scion. Error bars represent standard error of the mean (*n* = 3), and significant differences are shown in different letters (*p* < 0.05)

We have previously shown that in avocado, similar to additional species [15, 16, 24], high miR156 and low miR172 abundance in leaves is significantly associated with juvenility of the plant [17]. Thus, to predict the maturity/phase of the pre-grafting material, rootstock and scion leaf samples were analysed for miR156 and miR172 abundance. Consistent with their more juvenile state, seedling rootstocks had a significantly higher level of miR156 relative to clonal rootstocks (Fig. 1a), while miR172 showed the inverse expression pattern (Fig. 1b). Moreover, the expression of both these miRNAs in the clonal rootstocks perfectly mirrored that of the mature ‘Hass’ scion material taken directly from field trees (Fig. 1a, b). This suggests that clonal rootstocks of avocado retain the molecular maturity of their parent trees, despite undergoing the clonal rootstock production process.

To further explore this hypothesis, the expression of the miR156 and miR172 putative target genes were profiled. The abundance level of the predicted miR156 target, *PaSPL4*, was inversely correlated to miR156 and was highest in clonal rootstocks and budwood scions along with miR172 (Fig. 1a, c). *SPL4* is known to promote the transition to reproductive maturation in plants at least in part through activation of various floral identity genes [25–27]. miR172 acts antagonistically to miR156 and is considered a positive regulator of flowering by repressing *AP2 like* floral repressor genes [24, 28–30]. Transcript abundance of the *AP2* homolog, *PaRAP2.7B*, was inversely correlated with miR172 and highly expressed in the seedling rootstocks and scions relative to clonal rootstocks and budwood scions (Fig. 1b, d), consistent with its putative action as a floral repressor [13, 29, 31]. Taken together, the expression data of markers for juvenility and flowering suggest that seedling rootstocks and scions of avocado are in juvenile phase and clonal rootstocks and budwood are in adult or reproductive phase.

### Post-grafting: graft signalling and maturity in avocado

**Fig. 2:**
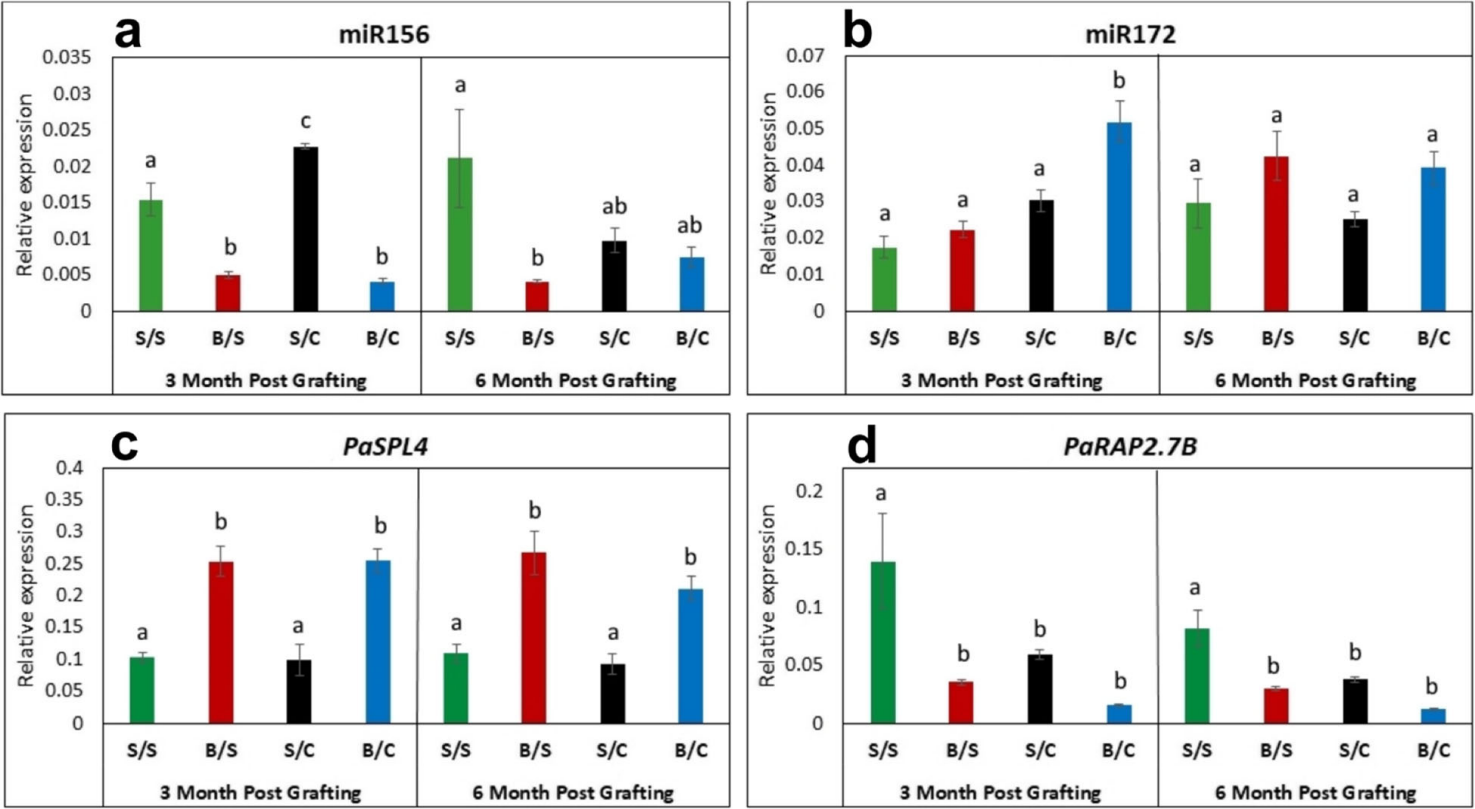
Expression profile of miRNAs and their putative target genes in avocado post-graft material. Expression analysis (qRT-PCR) of **a** miR156, **b** miR172, **c** *PaSPL4* and **d** *PaRAP2.7B* in post-grafting avocado leaves, ‘Hass’ as scion and ‘Velvick’ as rootstock. Where, S/S = seedling scion on seedling rootstock, B/S = budwood scion on seedling rootstock, S/C = seedling scion on clonal rootstock and B/C = budwood scion on clonal rootstock. Error bars represent standard error of the mean (n = 3), and significant differences are shown in different letters for 3 month and 6 months post-grafting calculated using one way ANOVA for each time-point, respectively. (p < 0.05)

To determine any possible effect of rootstock maturity and miRNA expression on the post-grafting scion, the youngest fully expanded leaves from the scion of the grafted plants were sampled 3 months and 6 months post-grafting, and then profiled for mature miR156 (Fig. 2a), miR172 (Fig. 2b), and their putative target genes (Fig. 2c, d). In 3 months post-grafted trees, miR156 abundance appeared to be governed by the scion (Fig. 2a). Budwood scions, which exhibit flowering during next flowering season, retained their low miR156 abundance, while the seedling scions (no flowering phenotype) showed high miR156 expression. This trend remained similar for 6 months samples, however in this case the results were confounded by lack of significance between samples – although miR156 in seedling scions was reduced to budwood levels when grafted to clonal rootstocks, this reduction was not significant compared to seedling/seedling grafts.

We then profiled *PaSPL4* to see if miR156 expression pattern can be translated into the target effect. Here, again the results suggest that the scion is primarily governing *PaSPL4* transcript abundance, as with miR156 (Fig. 2c). *PaSPL4* transcript abundance at 3 months post-grafting showed a strong negative correlation with miR156 abundance, where high transcript abundance was observed in budwood scions and vice versa in seedling scions (Fig. 2c). However, unlike for miR156, at 6 months post-grafting, *PaSPL4* expression was significantly scion dependent. This suggests a possibility of local regulation of *PaSPL4* independent of direct miR156 dependent age pathway.

Interestingly, there was a difference in miR172 expression between budwood scions grafted to seedling vs. clonal rootstocks at 3 months post-grafting. The budwood grafted onto the seedling rootstocks had similarly low miR172 expression pattern as seedling scions grafted on both seedling and clonal rootstocks. On the other hand, budwood grafted on clonal rootstocks had significantly higher abundance of miR172 (*p* < 0.05). This observed lower expression of miR172 using seedling rootstock suggests that rootstock may also have a role in determining grafted plant miR172 levels and potentially fate (Fig. 2b). However, this effect was absent 6 months post-grafting, where budwood grafted on seedling and clonal rootstocks both had similar expression patterns (Fig. 2b). In general, the expression pattern of *PaRAP2.7B* anticorrelated with the level of miR172 and was affected by rootstock type (Fig. 2d).

We also profiled the additional putative miR156 target genes, *PaSPL9a, PaSPL9b,* which are putative homologues of *SPL9* (avocado has two copies of *SPL9*), as well as *PaAP2* and *PaRAP2.7A*, which are putative miR172 targets. However, no clear pattern was observed for these transcripts depending on maturity of grafting material (Additional file 1: Figure S1 and S2), which is consistent with our previous finding suggesting these transcripts may not be involved in the maturity pathway in the leaves [17]. This observation may suggest a lack of miR156-*SPL9* and miR172-*AP2-Like* transcriptional regulatory module in grafted avocado leaves. These observations are based on mRNA transcript abundance and do not rule out a possibility of translational/posttranslational regulation of SPL9 and AP2-like proteins by miR156 and miR172, respectively. Another possibly of this disconnect can be the regulation of these genes independent of their respective miRNAs regulatory module.

Graft success, height of the tree and flowering data were collected for grafted plants. Greater than 80% graft success was observed for mature budwood scions grafted onto seedling as well as clonal rootstocks (commercial industry practice). Lower grafting success was observed when using seedling scions (Additional file 1: Table S1). Trees produced using clonal rootstocks were taller than those on seedling rootstocks (Mean height (cm) value with Standard Deviation (SD): budwood scion/ seedling rootstock = 46.5 cm ± 4.7, seedling scion/seedling rootstock = 43.5 cm ± 7.8, budwood scion/clonal rootstock = 92.8 cm ± 6.6 and seedling scion/clonal rootstock = 88.6 cm ± 9.6) (Additional file 1: Table S2). Flowering data correlated to the miRNA/target gene expression patterns in terms of maturity, where the budwood scions flowered the very next season from the time of grafting. On the other hand, seedling scions did not produce flowers on both rootstocks (Additional file 1: Table S2).

### Leaves as a potential source of signalling across the graft junction

Commercially, to achieve higher graft success, the leaves on clonal rootstocks are generally not removed during grafting as they are presumed to provide a source of energy for graft healing. Meanwhile, seedling rootstocks are devoid of leaves because the growing shoot tip with leaves is removed, and presumably graft healing is promoted by energy remaining from the seed. As the presence of leaves is a major difference between clonal and seedling rootstocks, we hypothesized that the leaves may contribute not only to graft health, but to intergraft signalling. Thus, to check whether leaves have any effect on the apparent inter-graft signalling noted for miR172 in clonal grafts we designed an independent experiment where ‘Hass’ budwood was grafted onto ‘Velvick’ clonal rootstocks in two groups; one group with leaves left on the rootstock (with leaves ‘WL’ grafts), the other group where leaves were removed from the rootstock (without leaves ‘WOL’ grafts). We then profiled miR156 and miR172 in the scions of both graft combinations 3 months post-grafting. We also profiled the abundance of *PaTPS1*, which has been shown to indicate carbohydrate availability in plants [22], as marker for possible rootstock-derived carbohydrates moving to the scion.

**Fig. 3:**
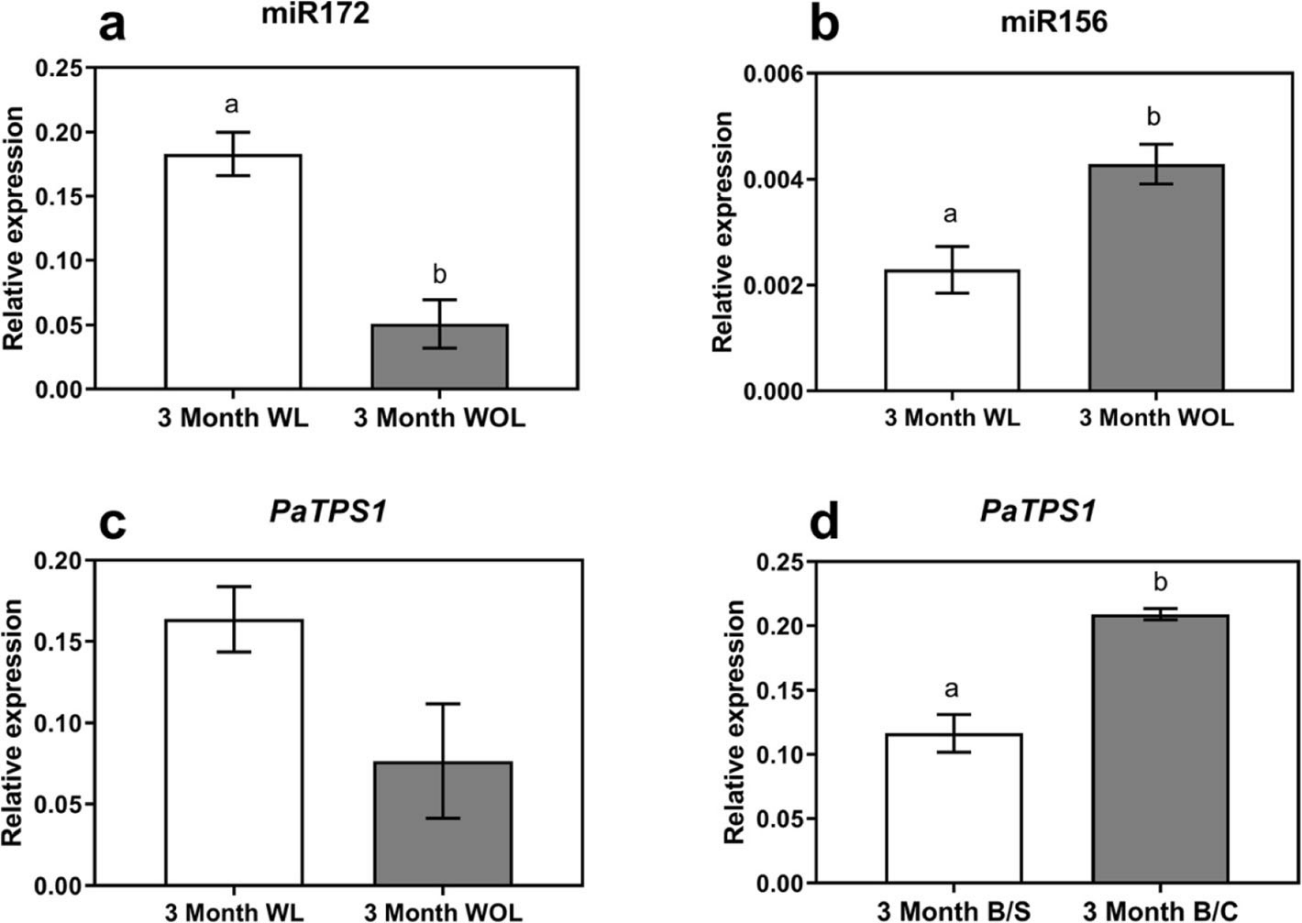
miR156/miR172/*PaTPS1* transcript abundance corresponds to the presence/absence of leaves in the rootstock. Expression analysis (qRT-PCR) to compare effect of leaves on grafted tree molecular profiles of **a** miR172, **b** miR156 and **c** *PaTPS1* in post-grafting avocado leaves, ‘Hass’ budwood scion and ‘Velvick’ as clonal rootstock in two group; with leaves and without leaves, **d** *PaTPS1* expression in 3-month post-grafting comparison in budwood on seedling and budwood on clonal. Error bars represent standard error of the mean (*n* = 3), different letter show significant difference (*p* < 0.05)

Consistent with industry know-how, the graft success rate was more than 80% for the grafts with leaves while it was less than 30% when leaves were removed from the clonal rootstock prior to grafting (Additional file 1: Table S1). Moreover, consistent with our hypothesis that the leaves contributed to the higher miR172 level in B/C grafts relative to B/S grafts above, significantly higher miR172 abundance was observed in scions sampled from grafts with leaves, as compared to the grafts where leaves were removed (Fig. 3a). Opposite trends were observed for miR156 abundance, which was significantly higher in grafts where leaves were removed from rootstocks (Fig. 3b). Similarly, to miR172, *PaTPS1* abundance in the scion was higher in the grafts where the rootstock leaves were not removed (Fig. 3c, d). This suggests a possible role for leaves in inter-graft signalling of miR156, miR172 in avocado, and which may involve mobile sugars.

## Discussion

Grafting is widely used as the preferred method of propagation in horticultural trees, which combines a desirable scion with a suitable rootstock cultivar to improve productivity outcomes. In avocado, scion material comprises budwood collected from mature trees of a fruiting cultivar, while rootstocks may either be derived from seed or from mature tree cuttings. Although more difficult to produce, the use of rooted cuttings provides genetic uniformity of the rootstock for these outcrossing species.

Field observation has shown that a major benefit of grafting is the reduction of the juvenile phase in the grafted tree, leading to earlier productivity. However, the molecular players involved, and relative impact of grafting material maturity on the grafted tree maturity remains unknown. We have previously shown that the phase transition related miRNAs miR156, miR172, and the miR156 target gene *SPL4*, can be reliably used as markers to differentiate between juvenile and adult trees/material in avocado [17]. Graft transmissible signalling of miR156 and miR172 has been reported in potato [9, 10]. This makes these miRNAs ideal candidates to explore whether rootstocks derived from seedlings or mature cuttings show different molecular maturity profiles, and if this may have any influence on the scion maturity profile. Here, we employed a molecular strategy to investigate the maturity of pre-graft and post-graft avocado trees, using both seedling and cutting rootstocks.

Generally, avocado seedlings can be used as rootstocks for grafting as young as two months old. On the other hand, preparation of clonal rootstocks requires use of a specialised technique to induce root formation on the cutting/budwood taken from a mature tree. However, it is still unknown whether these clonal rootstocks maintain the same maturity level as their source tree after going through the rooting process.

Using the miR156-*SPL4*/miR172 model of juvenility and phase transition, here we examined the maturity level of the pre-graft materials in addition to the grafted plant at the time of typical commercial supply. Avocado seedling scions and rootstocks showed a juvenile miRNA profile compared to the budwood taken from a mature tree: seedlings showed a high abundance of miR156 while budwood material had a low abundance, and vice versa for its downstream targets *PaSPL4* and miR172. This aligns with previous findings in avocado [17] and other crops [13, 15] where a similar molecular profile was associated to juvenile and adult plants. We further observed that avocado clonal rootstocks have a similar miRNA profile to the budwood material, with low miR156 and high miR172 abundance, and negative correlation to their target transcripts (*PaSPL4* and *PaRAP2.7A*) (Fig. 1). This is consistent with the roles of these genes in other species and suggests that even though these clonal rootstock plants were subjected to an intense rooting process they retained the maturity level of their source trees.

Previously, it has been suggested that miR156 and miR172 are graft transmissible miRNAs in potato [9, 10]. The question here is whether genetically different parts of the grafted tree (scion and rootstock) can interchange molecular signals involved in juvenility. In order to profile maturity-related miRNAs of avocado trees post-grafting and detect any possible inter-graft signalling from rootstocks of different maturity, we evaluated post-graft samples for miR156 and miR172 abundance and target gene effects. Consistent with the role of miR156 in juvenility promotion in plants [12, 13, 15, 24], miR156 abundance in avocado seedling scions remained significantly greater than in budwood scions 3 months post-grafting. In addition, an anti-correlative association was observed between miR156 and its predicted target gene *PaSPL4* at 3 months. By 6 months post grafting, mature miR156 abundance in seedling scions grafted on clonal rootstock plants was reduced to budwood levels, suggesting a rootstock effect. However, this reduction was not significant relative to seedling on seedling grafts, thus a clear effect of rootstock on seedling scion miR156 level could not be confirmed. Similarly, for budwood scions, neither miR156 nor *PaSPL4* were affected by rootstock maturity (seedling vs clonal). Thus, despite the difference in miR156 and *PaSPL4* abundance between the two rootstock types pre-grafting, there appears to be no significant transmissibility of this status to the scions. Moreover, consistent with a functioning miR156-*SPL4* regulatory module in avocado, the levels of miR156 and *SPL4* were generally anticorrelated, although this was less clear 6 months post-grafting where miR156 expression declined. This may suggest a possibility of *SPL4* regulation independent of the miR156 dependent age regulatory pathway in avocado as was observed in *Arabidopsis* where *SPL3/4/5* were regulated independent of miR156-dependent age pathway [22].

Commercially propagated avocado plants comprising budwood scions grafted on either seedling or clonal rootstocks are observed to acquire floral competency in very next season after field planting. However, this phenomenon has not been quantified in the literature to our best knowledge. Grafts using mature scionwood produced flowers the next flowering season after grafting. Meanwhile, grafts with seedling scions did not produce flowers regardless of rootstock. This aligns with field observation and the molecular data showing primarily scion control of juvenility associated genes discussed above.

Nonetheless, here we provide some limited evidence for inter-graft regulation of miR156 and miR172 in avocado in at least one grafting combination and timepoint. At 3 months post-grafting, the expression of miR172 in budwood scions was affected by rootstock, with levels reduced to those seen for seedling scions specifically in budwood grafted on seedling rootstocks. This may provide evidence for a possible repressor of miR172 moving from the seedling rootstock to the mature scion, or a promoter of miR172 moving from the clonal rootstock. An obvious candidate for a negative regulator would be a mobile miR156, or miR156-regulated, signal, given that miR156 is indirect negative regulator of miR172 in *Arabidopsis* and other plants [13, 15, 24, 32]. However, we saw no evidence for increased miR156 abundance in budwood scions grafted on seedling rootstocks at this time (Fig. 2a). Ultimately, by 6 months post-grafting, the levels of miR172 in budwood scions was equivalent between seedling and clonal grafts. This suggests that even though there may be early graft-transmissible effects, miRNA level in the scion largely depends on the scion material.

**Fig. 4:**
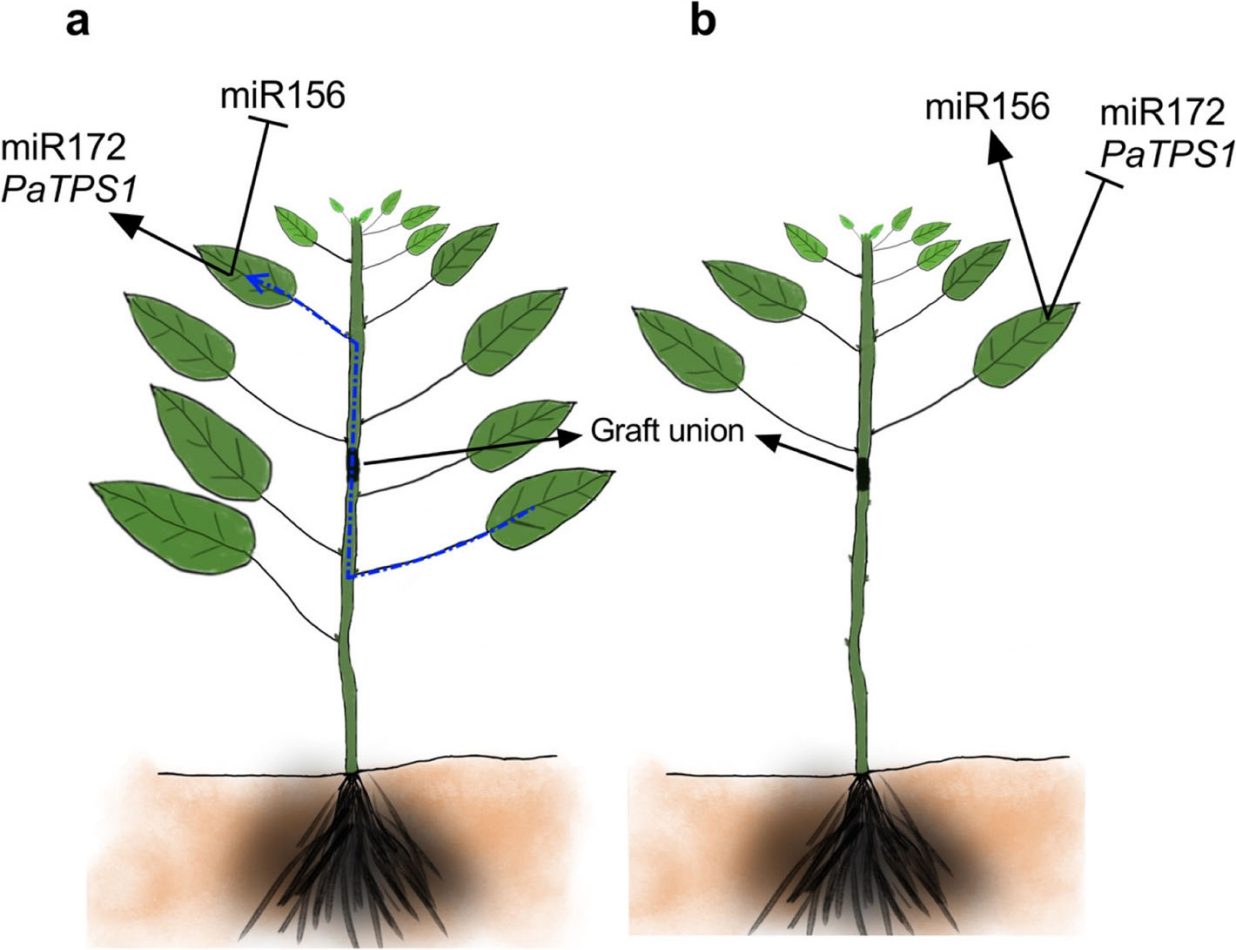
Proposed inter-graft signalling in 3 month-post grafted avocado trees - leaves on the rootstock contribute to graft-signalling. Intact leaves in the rootstock affect transcript abundance in the scion: **a** avocado grafts with leaves on rootstock, where miR172 and *PaTPS1* were upregulated. On the other hand, removing leaves results in an increase in miR156 abundance and downregulation of miR172/*PaTPS1* in the scion **b** avocado grafts without leaves on clonal rootstock. This suggests a possibility of leaf-regulated graft transmissibility of signals affecting miR156, miR172 and *TPS1* transcript abundance in the scion

In a commercial nursery setting, the leaves are normally left on clonal rootstocks as these leaves are considered critical for graft success. Seedling rootstocks lack leaves however they are still fed from the cotyledonary energy stores, thought to help with graft take. Generally, leaves are known to be involved in multidirectional long-distance signalling in plants; towards both root and apical meristems [33–35]. The movement of molecular factors from leaves to the apical meristem has already been established [34]. We hypothesized that the presence of leaves specifically on clonal rootstocks could be a differentiating factor, unrelated to maturity, affecting rootstock-dependent gene expression in the scion. Here we reveal that removing leaves from clonal rootstocks does influence miR156 and miR172 abundance in the scion (Fig. 4). Leaves are the powerhouse of the plants and the main source of energy/photosynthate production. To further investigate whether the carbohydrate availability of the plant may contribute in this scenario we profiled the carbohydrate availability marker *TPS1* (a key enzyme in T6P pathway) which is known to interact with age-dependent flowering pathway upstream of miR156 [21]. We show that *PaTPS1* transcript abundance in the scion leaves decreased in grafts where leaves were removed from the rootstock (Fig. 4b). This suggests that leaves below the graft union contribute to sugar signalling in the scion in these species. In this way, removing leaves likely impacts the carbohydrate availability to the plants supporting the commercial industry practice to retain leaves to increase graft success. This is further evident from our observation that graft success in our hands was less than 30% in grafts where leaves were removed from rootstocks.

## Conclusions

In conclusion, here we suggest that grafted tree maturity is governed largely by the scion in avocado. However, we provide some evidence for graft transmissible regulation of miR156 and miR172, which may involve leaves below the graft union. Our findings are consistent with industry observations of grafted tree maturity and for the first time have started to explain the underlying molecular mechanisms involved. This information will help to better understand bottlenecks in grafting, graft success and propagation of horticultural trees, where type of scion and rootstock material greatly effect propagation success and operational costs.

## Methods

### Plant material and grafting

**Fig. 5:**
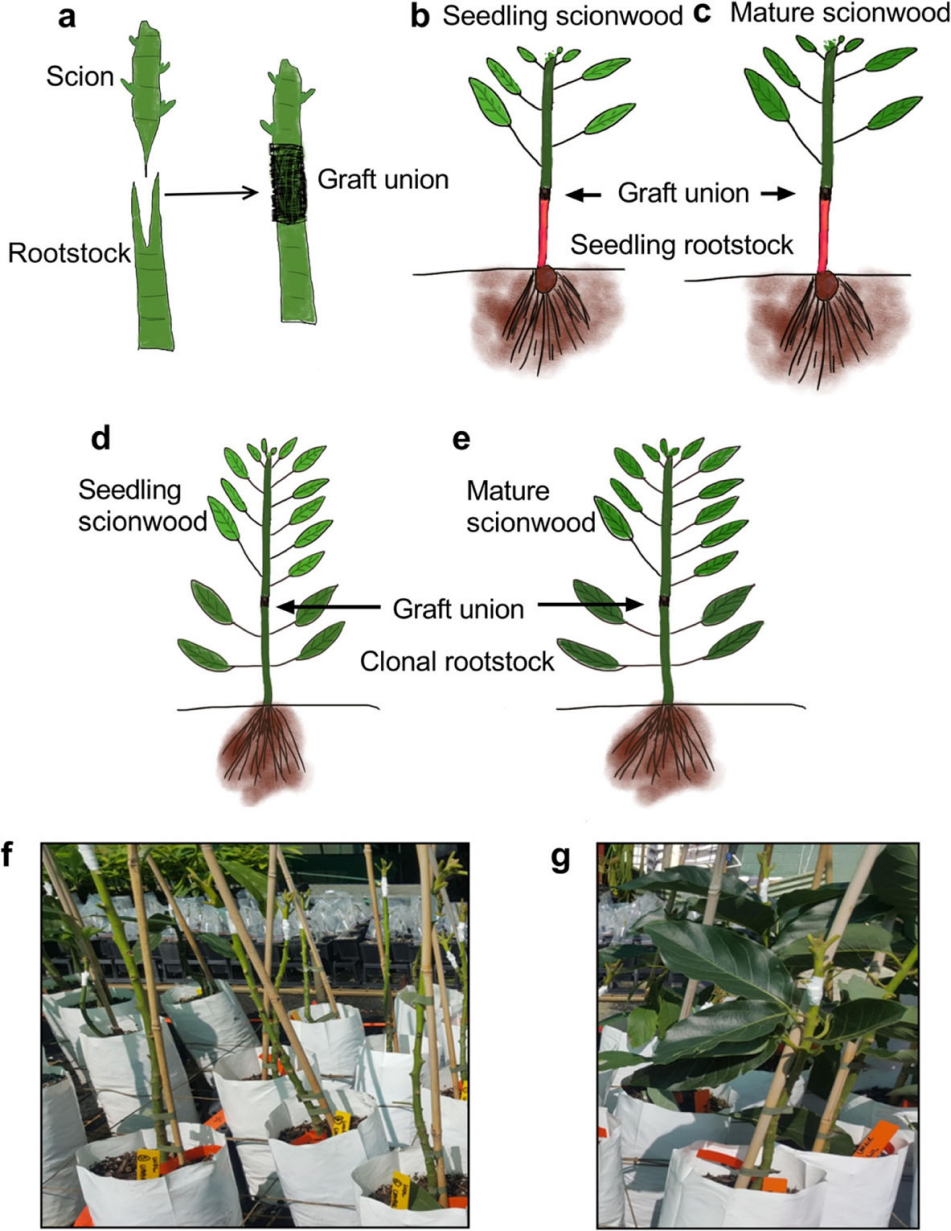
Grafting techniques and combinations used. Schematic diagrams of a Wedge grafting in avocado. Simplified graphical representations of avocado graft combinations 3 months post-grafting: **b** seedling scionwood grafted on seedling rootstock, **c** mature scionwood grafted on seedling rootstock, **d** seedling scionwood grafted on clonal rootstock and **e** mature scionwood grafted on clonal rootstock. Photographs of avocado defoliation experiment combinations on the day of grafting: **f** grafts without leaves on rootstock, **g** grafts with leaves on rootstock

Avocado cv. ‘Hass’ and cv. ‘Velvick’ seeds and propagation material were provided by Anderson Horticulture Pty Ltd. and grown in Anderson nursery located in Duranbah, New South Wales, Australia. All avocado grafting experiments were generated at Anderson Horticulture Pty Ltd. nursery (appropriate permissions were obtained). Approx. 2 months old seedlings of cultivars ‘Velvick’ and ‘Hass’ were generated for seedling rootstocks and scions respectively. Clonal rootstocks of ‘Velvick’ were prepared using the Frolich method modified from Ernst, 1999 [36, 37], and ‘Hass’ mature scionwood (budwood) for mature scions was collected from adult trees as per commercial practise at Anderson Horticulture. Wedge grafts (Fig. 5a) were made in four possible combinations of 12 plants (scion/rootstock) (Fig. 5b-e): 1) budwood ‘Hass’/seedling ‘Velvick’, 2) budwood ‘Hass’/clonal ‘Velvick’, 3) seedling ‘Hass’/seedling ‘Velvick’, and 4) seedling ‘Hass’/clonal ‘Velvick’. Leaf samples were collected immediately pre-grafting and at 3 months and 6 months post-grafting. Another independent experiment was designed to determine the effect of clonal rootstock leaves on inter-graft signalling. Here budwood ‘Hass’ was grafted on ‘Velvick’ clonal rootstocks in two groups of 12 plants; 1) ‘With Leaves (WL)’ - where leaves were left on the rootstock as per conventional practice, 2) ‘Without Leaves (WOL)’ - where leaves were removed from the rootstock (Fig. 5f, g). Leaves for expression analysis were sampled 3 months post-grafting. In all cases, the youngest fully expanded leaf was sampled and pooled into three biological replicates directly on dry ice and stored at -80 °C freezer.

### RNA extraction

Leaf tissues were ground to fine powder under liquid nitrogen and total RNA was extracted using a MasterPure Plant RNA Purification kit (Epicentre, USA). RNA was quantified using a Nanodrop ND-1000 spectrophotometer and quality assessed on 1% TAE agarose gels. For miRNA quantification, 500 ng total RNA was utilized for low-molecular weight cDNA synthesis (to quantify mature miRNAs) with a miScript Plant RT Kit (Qiagen, Netherlands). For gene quantification, 600 ng of total RNA was used to synthesize high molecular weight cDNA using a SensiFAST™ cDNA Synthesis Kit (Bioline, Australia).

### Quantitative real time polymerase chain reaction

#### miRNA quantification

qRT-PCR reactions were performed in duplicate for each biological replicate using a miScript SYBR® Green PCR Kit (cDNA prepared using a miScript Plant RT Kit was utilized) (QIAGEN, The Netherland). The primer sequences of miRNAs (miR156 and miR172) and housekeeping transcripts (U6 [38], 5.8S rRNA [39]) are shown in Additional file 1: Table S3. Reactions were performed on a Rotor-Gene Q 6000 (Qiagen, The Netherlands) and visualized using Rotor-Gene Q 2.3.1.49 software (QIAGEN, The Netherland).

#### Gene quantification

Primer sequences from our recent manuscript [17] were utilized for gene quantification (Additional file 1: Table S3) using a SensiFAST™ SYBR® No-ROX Kit (Bioline, Australia) in a BioRad CFX384 Touch™ Real-Time PCR Detection System (Bio-Rad, USA) in accordance with manufacturer instructions and visualized on CFX Manager™ Software (Bio-Rad, USA).

For all qPCR runs, PCR efficiencies were computed using LinReg PCR version 7.5 (University of Amsterdam, Netherlands). This Data was then further analysed and evaluated to determine the relative abundance of miRNA and genes by employing the formula:

$$ \mathrm{Relative}\ \mathrm{abundance}/\mathrm{expression}=\mathrm{Gene}\ \mathrm{PE}\hat{\mkern6mu} \left(-\mathrm{Gene}\ \mathrm{Ct}\right)/\mathrm{Control}\ \mathrm{PE}\hat{\mkern6mu} \left(-\mathrm{Control}\ \mathrm{Ct}\right) $$

where PE is primer efficiency and Ct is cycle threshold of each reaction. To check statistical significance of the data, a one-way analysis of variance (ANOVA) was done with Tukey correction (a post-hoc multiple comparison tests to compare means) using SPSS version 23 (IBM, USA). The averages of relative expression of miRNAs and genes were plotted with standard error using GraphPad Prism 6 (GraphPad Software Inc.).

## Additional file

Additional file 1: **Figure S1.** Expression analysis of a *PaSPL9a*, b *PaSPL9b*, c *PaRAP2.7A* and d *PaAP2* in pre-graft avocado leaves sampled from the seedling rootstock, clonal rootstock, seedling scion and budwood scion. Error bars represent standard error of the mean (*n* = 3), and significant differences are shown in different letters (*p* < 0.05). **Figure S2.** Expression analysis of a *PaSPL9a*, b *PaSPL9b*, c *PaRAP2.7A* and d *PaAP2* in post grafting avocado leaves, cv. Hass as scion and cv. Velvick as rootstock. Where, S/S = seedling scion on seedling rootstock, B/S = budwood scion on seedling rootstock, S/C = seedling scion on clonal rootstock and B/C = budwood scion on clonal rootstock. Error bars represent standard error of the mean (n = 3). **Table S1.** Graft success observed for avocado grafts. **Table S2.** Phenotypic observation for flowering and plant height in the avocado grafted plants. Where, Hb = Budwood Hass, Hs = Seedling Hass used as scion, Vs = Velvick seedling rootstock and Vc = Velvick Clonal rootstock. **Table S3.** Primers used for miRNA and gene quantification. (DOCX 432 kb)

## Data Availability

The datasets used and/or analysed during the current study are available from the corresponding author on reasonable request.

## Abbreviations

miRNA: microRNA
qRT-PCR: Quantitative Real-Time Polymerase Chain Reaction
*SPL*: *SQUAMOSA PROMOTER BINDING PROTEIN-LIKE*
WL: With Leaves
WOL: Without Leaves
