# Supplementary material for: Scion control of miRNA abundance and tree maturity in grafted avocado
Source: BMC Plant Biol. 2019 Sep 3;19:382. doi: 10.1186/s12870-019-1994-5 (PMC6724330; doi:10.1186/s12870-019-1994-5)
Supplement: Supplementary file 1 — Figure S1. Expression analysis of a PaSPL9a, b PaSPL9b, c PaRAP2.7A and d PaAP2 in pre-graft avocado leaves sampled from the seedling rootstock, clonal rootstock, seedling scion and budwood scion. Error bars represent standard error of the mean (n = 3), and significant differences are shown in different letters (p < 0.05). Figure S2. Expression analysis of a PaSPL9a, b PaSPL9b, c PaRAP2.7A and d PaAP2 in post grafting avocado leaves, cv. Hass as scion and cv. Velvick as rootstock. Where, S/S = seedling scion on seedling rootstock, B/S = budwood scion on seedling rootstock, S/C = seedling scion on clonal rootstock and B/C = budwood scion on clonal rootstock. Error bars represent standard error of the mean (n = 3). Table S1. Graft success observed for avocado grafts. Table S2. Phenotypic observation for flowering and plant height in the avocado grafted plants. Where, Hb = Budwood Hass, Hs = Seedling Hass used as scion, Vs = Velvick seedling rootstock and Vc = Velvick Clonal rootstock. Table S3. Primers used for miRNA and gene quantification. (DOCX 432 kb) [file 12870_2019_1994_MOESM1_ESM.docx]

## Additional file 1:


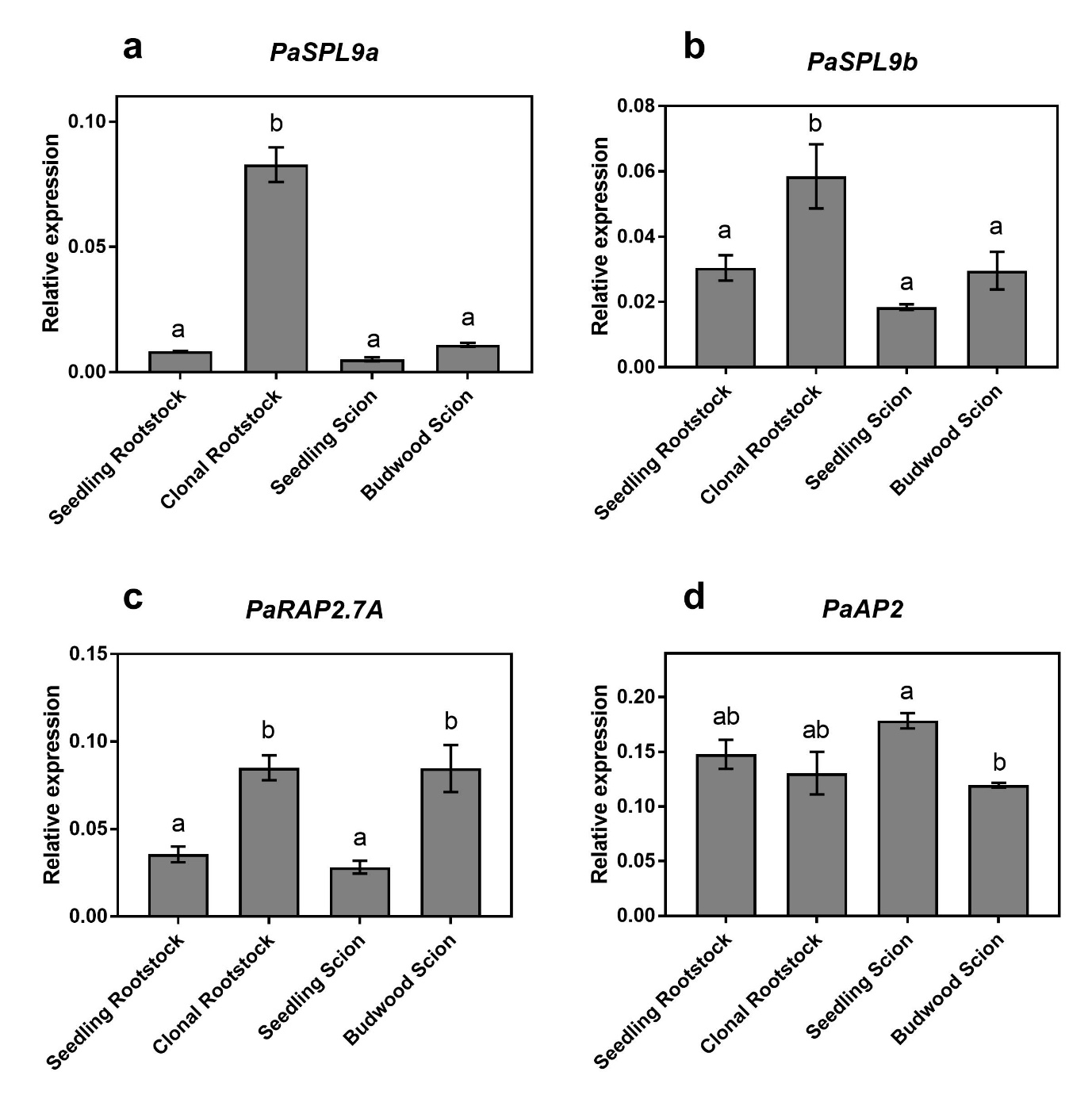


Additional file 1: Fig. S1: Expression analysis of a *PaSPL9a*, b *PaSPL9b*, c *PaRAP2.7A* and d *PaAP2* in pre-graft avocado leaves sampled from the seedling rootstock, clonal rootstock, seedling scion and budwood scion. Error bars represent standard error of the mean (n=3), and significant differences are shown in different letters (p<0.05).


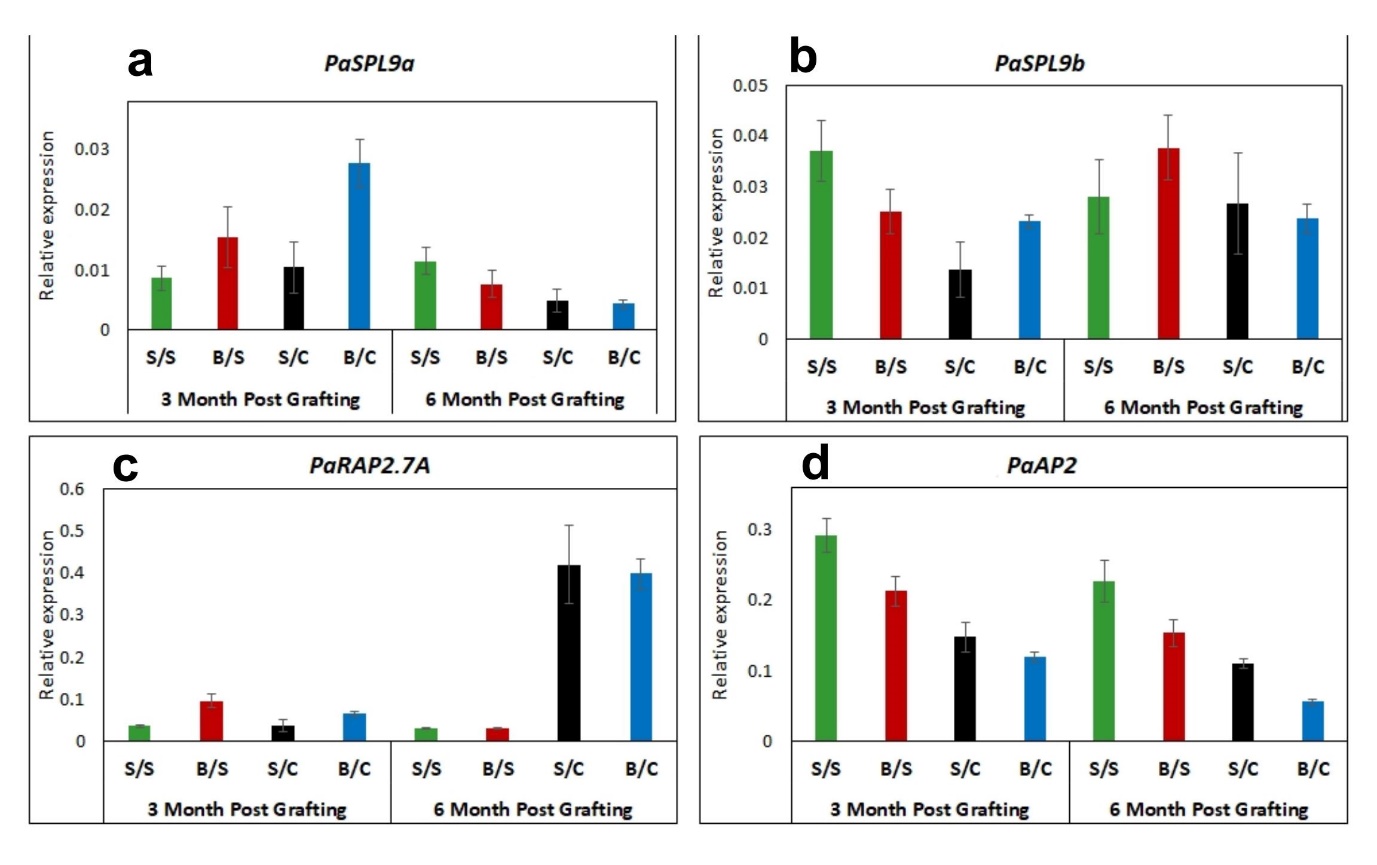


Additional file 1: Fig. S2: Expression analysis of a *PaSPL9a*, b *PaSPL9b*, c *PaRAP2.7A* and d *PaAP2* in post grafting avocado leaves, cv. Hass as scion and cv. Velvick as rootstock. Where, S/S= seedling scion on seedling rootstock, B/S= budwood scion on seedling rootstock, S/C= seedling scion on clonal rootstock and B/C= budwood scion on clonal rootstock. Error bars represent standard error of the mean (n=3).

Additional file 1: Table S1: Graft success observed for avocado grafts.

| **1** | **Graft combination** | **Total grafts** | **Success** | **Died** | **Graft success %** |
| --- | --- | --- | --- | --- | --- |
|  | Budwood Hass/ Seedling Velvick | 22 | 20 | 2 | 90.90909091 |
|  | Seedling Hass/ Seedling Velvick | 21 | 12 | 9 | 57.14285714 |
|  | Budwood Hass/ Clonal Velvick | 12 | 10 | 2 | 83.33333333 |
|  | Seedling Hass/ Clonal Velvick | 12 | 5 | 7 | 41.66666667 |
|  |  |  |  |  |  |
| **2** | **Leaf removal vs intact leaves in rootstock part experiment** | **Total grafts** | **Success** | **Died** | **Graft success %** |
|  | Budwood Hass/ Clonal Velvick with intact leaves | 12 | 10 | 2 | 83.33333333 |
|  | Budwood Hass/ Clonal Velvick Without leaves | 12 | 3 | 9 | 25 |

Additional file 1: Table S2: Phenotypic observation for flowering and plant height in the avocado grafted plants. Where, Hb= Budwood Hass, Hs= Seedling Hass used as scion, Vs= Velvick seedling rootstock and Vc= Velvick Clonal rootstock.

| Graft combination | sample no | Flowering- (July – 8-months post-grafting) | Height (cm) 6 months post-grafting |
| --- | --- | --- | --- |
| Hb/Vs | 1 | yes | 45 |
| Hb/Vs | 2 | yes | 48 |
| Hb/Vs | 3 | yes | 40 |
| Hb/Vs | 4 | yes | 52 |
| Hb/Vs | 5 | yes | 53 |
| Hb/Vs | 6 | yes | 50 |
| Hb/Vs | 7 | yes | 44 |
| Hb/Vs | 8 | yes | 49 |
| Hb/Vs | 9 | yes | 45 |
| Hb/Vs | 10 | yes | 39 |
| Hs/Vs | 1 | No | 44 |
| Hs/Vs | 2 | No | 51 |
| Hs/Vs | 3 | No | 28 |
| Hs/Vs | 4 | No | 39 |
| Hs/Vs | 5 | No | 42 |
| Hs/Vs | 6 | No | 54 |
| Hs/Vs | 7 | No | 37 |
| Hs/Vs | 8 | No | 50 |
| Hs/Vs | 9 | No | 41 |
| Hs/Vs | 10 | No | 49 |
| Hb/Vc | 1 | Yes | 95 |
| Hb/Vc | 2 | Yes | 100 |
| Hb/Vc | 3 | Yes | 93 |
| Hb/Vc | 4 | Yes | 85 |
| Hb/Vc | 5 | Yes | 93 |
| Hb/Vc | 6 | Yes | 98 |
| Hb/Vc | 7 | Yes | 90 |
| Hb/Vc | 8 | Yes | 81 |
| Hb/Vc | 9 | Yes | 101 |
| Hs/Vs | 1 | No | 93 |
| Hs/Vs | 2 | No | 100 |
| Hs/Vs | 3 | No | 90 |
| Hs/Vs | 4 | No | 86 |
| Hs/Vs | 5 | No | 74 |

Additional file 1: Table S3: Primers used for miRNA and gene quantification.

| **No.** | **Gene** | **F Primer** | **R Primer** |
| --- | --- | --- | --- |
| 1 | miR156 | TGACAGAAGAGAGTGAGCAC | Universal Primer |
| 2 | miR172 | AGAATCTTGATGATGCTGCAT | Universal Primer |
| 3 | U6 SnoRNA | GGATGACACGCACAAATCGAG | Universal Primer |
| 5 | 5.8S ribosomal RNA rRNA | GAATTGCAGAATCCCGTGAACC | Universal Primer |
| 6 | *PaRAP2.7A/TOE1A* | GCAGTAGAGAGGGCTGAACTC | GTAACAGTAGGTAGGAAAATGGCC |
| 7 | *PaRAP2.7B* | CAACTACGAGGCTCTACTGC | GCTTTCAGCTCTGGTAATAGTAGG |
| 8 | *PaAP2* | GGAGCAGTAGTAATTGGGCTGC | CATAATCAAGGGTCAGGTAGGTC |
| 9 | *PaSPL4* | CGTTGGTTCTCTACTATGCTCTC | GTACCTACTGGCACAGATAGC |
| 10 | *PaSPL9a* | CTTTGGCAGGGTAGCATAGAG | GCTGTGATACCCGAAGCTCG |
| 11 | *PaSPL9b* | GCAGGAATCTCCAACTCCAG | TGGCACTATGACTTGGTGGA |
| 12 | *PaTPS1* | CTTCAAGCGAGGGATATGCT | TGCTGCACCCTTTGTAACAC |
| 13 | *PaGAPDH* | TGGGAAACTTACAGGAATGG | GTCACCCACAAAGTCAGTAGAA |
| 14 | *PaEF1a* | ATCAAGCGTGGGTTTGTTGC | TACCCGTTGCCAATCTGACC |
